# American Medical Times Suspended

**Published:** 1864-09

**Authors:** 


*American Medical Times suspended.*—The issue of the *American Medical Times* is temporarily suspended. The publishers announce that they are compelled to this by the enormous rise and constant increase of every thing relating to publication. All subscribers who have paid in advance are informed that the amount due them will be refunded on presentation of their receipts, if they are unwilling to let it remain until the publication is again commenced. Messrs. Bailliere Bros. hope before many months to be enabled to resume its publication.

